# Brain Temperature Influences Intracranial Pressure and Cerebral Perfusion Pressure After Traumatic Brain Injury: A CENTER-TBI Study

**DOI:** 10.1007/s12028-021-01294-1

**Published:** 2021-07-30

**Authors:** Tatiana Birg, Fabrizio Ortolano, Eveline J. A. Wiegers, Peter Smielewski, Yan Savchenko, Bogdan A. Ianosi, Raimund Helbok, Sandra Rossi, Marco Carbonara, Tommaso Zoerle, Nino Stocchetti, the CENTER-TBI Investigators and Participants, Audny Anke, Ronny Beer, Bo-Michael Bellander, Erta Beqiri, Andras Buki, Manuel Cabeleira, Arturo Chieregato, Giuseppe Citerio, Hans Clusmann, Endre Czeiter, Marek Czosnyka, Bart Depreitere, Ari Ercole, Shirin Frisvold, Stefan Jankowski, Danile Kondziella, Lars-Owe Koskinen, Ana Kowark, David K. Menon, Geert Meyfroidt, Kirsten Moeller, David Nelson, Anna Piippo-Karjalainen, Andreea Radoi, Arminas Ragauskas, Rahul Raj, Jonathan Rhodes, Saulius Rocka, Rolf Rossaint, Juan Sahuquillo, Oliver Sakowitz, Nina Sundström, Riikka Takala, Tomas Tamosuitis, Olli Tenovuo, Peter Vajkoczy, Alessia Vargiolu, Rimantas Vilcinis, Stefan Wolf, Alexander Younsi, Frederick A. Zeiler

**Affiliations:** 1 Neurological Intensive Care Unit, Fondazione IRCCS (Istituto di Ricovero e Cura a Carattere Scientifico) Ca’ Granda Ospedale Maggiore Policlinico, Milan, Italy; 2 Department of Public Health, Center for Medical Decision Sciences, Erasmus University Medical Center, Erasmus University, Rotterdam, Netherlands; 3 Brain Physics Lab, Division of Neurosurgery, Department of Clinical Neurosciences, Addenbrooke’s Hospital, University of Cambridge, Cambridge, UK; 4 Neurointensive Care Unit, Burdenko Neurosurgical Center, Moscow, Russia; 5 Neurological Intensive Care Unit, Department of Neurology, Medical University of Innsbruck, Innsbruck, Austria; 6 Department of Anesthesia and Intensive Care, Parma University Hospital, Parma, Italy; 7 Department of Pathophysiology and Transplant, Milan University, Milan, Italy; 8 Department of Physical Medicine and Rehabilitation, University Hospital Northern, Tromsø, Norway; 9 Department of Neurology, Neurological Intensive Care Unit, Medical University of Innsbruck, Innsbruck, Austria; 10 Department of Neurosurgery and Anesthesia and Intensive Care Medicine, Karolinska University Hospital, Stockholm, Sweden; 11 NeuroIntensive Care, Niguarda Hospital, Milan, Italy; 12 Department of Neurosurgery, Medical School, University of Pécs, Pécs, Hungary; 13 Neurotrauma Research Group, János Szentágothai Research Centre, University of Pécs, Pécs, Hungary; 14 Brain Physics Lab, Division of Neurosurgery, Department of Clinical Neurosciences, Addenbrooke’s Hospital, University of Cambridge, Cambridge, UK; 15 NeuroIntensive Care Unit, Department of Anesthesia and Intensive Care, ASST di Monza, Monza, Italy; 16 School of Medicine and Surgery, Università Milano Bicocca, Milan, Italy; 17 Department of Neurosurgery, Medical Faculty, RWTH Aachen University, Aachen, Germany; 18 Department of Neurosurgery, University of Pécs, Pécs, Hungary; 19 Hungarian Brain Research Program (Grant No. KTIA 13 NAP-A-II/8), MTA-PTE Clinical Neuroscience MR Research Group, János Szentágothai Research Centre, University of Pécs, Pécs, Hungary; 20 Department of Neurosurgery, University Hospitals Leuven, Leuven, Belgium; 21 Division of Anaesthesia, Addenbrooke’s Hospital, University of Cambridge, Cambridge, UK; 22 Department of Anesthesiology and Intensive Care, University Hospital Northern Norway, Tromso, Norway; 23 Neurointensive Care, Sheffield Teaching Hospitals NHS Foundation Trust, Sheffield, UK; 24 Departments of Neurology, Clinical Neurophysiology and Neuroanesthesiology, Region Hovedstaden Rigshospitalet, Copenhagen, Denmark; 25 Department of Clinical Neuroscience, Neurosurgery, Umeå University, Umeå, Sweden; 26 Department of Anaesthesiology, University Hospital of Aachen, Aachen, Germany; 27 Intensive Care Medicine, University Hospitals Leuven, Leuven, Belgium; 28 Department Neuroanesthesiology, Region Hovedstaden Rigshospitalet, Copenhagen, Denmark; 29 Helsinki University Central Hospital, Helsinki, Finland; 30 Department of Neurosurgery, Vall D’Hebron University Hospital, Barcelona, Spain; 31 Department of Neurosurgery, Kaunas University of Technology and Vilnius University, Vilnius, Lithuania; 32 Department of Anaesthesia, Critical Care and Pain Medicine NHS Lothian, University of Edinburg, Edinburgh, UK; 33 Klinik Für Neurochirurgie, Klinikum Ludwigsburg, Ludwigsburg, Germany; 34 Department of Neurosurgery, University Hospital Heidelberg, Heidelberg, Germany; 35 Department of Radiation Sciences, Biomedical Engineering, Umeå University, Umeå, Sweden; 36 Perioperative Services, Intensive Care Medicine, and Pain Management, Turku University Central Hospital, University of Turku, Turku, Finland; 37 Neuro-Intensive Care Unit, Kaunas University of Health Sciences, Kaunas, Lithuania; 38 Rehabilitation and Brain Trauma, Turku University Central Hospital, University of Turku, Turku, Finland; 39 Neurologie, Neurochirurgie und Psychiatrie, Charité – Universitätsmedizin Berlin, Berlin, Germany; 40 Department of Neurosurgery, Kaunas University of Health Sciences, Kaunas, Lithuania; 41 Department of Neurosurgery, Berlin Institute of Health, Charité – Universitätsmedizin Berlin, Corporate Member of Freie Universität Berlin, Humboldt-Universität Zu Berlin, Berlin, Germany; 42 Section of Neurosurgery, Department of Surgery, Rady Faculty of Health Sciences, University of Manitoba, Winnipeg, MB Canada

**Keywords:** Traumatic brain injury, Neuromonitoring, Brain temperature, Fever, Hyperthermia, Intracranial pressure, Cerebral perfusion pressure

## Abstract

**Background:**

After traumatic brain injury (TBI), fever is frequent. Brain temperature (BT), which is directly linked to body temperature, may influence brain physiology. Increased body and/or BT may cause secondary brain damage, with deleterious effects on intracranial pressure (ICP), cerebral perfusion pressure (CPP), and outcome.

**Methods:**

Collaborative European NeuroTrauma Effectiveness Research in Traumatic Brain Injury (CENTER-TBI), a prospective multicenter longitudinal study on TBI in Europe and Israel, includes a high resolution cohort of patients with data sampled at a high frequency (from 100 to 500 Hz). In this study, simultaneous BT, ICP, and CPP recordings were investigated. A mixed-effects linear model was used to examine the association between different BT levels and ICP. We additionally focused on changes in ICP and CPP during the episodes of BT changes (Δ BT ≥ 0.5 °C lasting from 15 min to 3 h) up or downward. The significance of ICP and CPP variations was estimated with the paired samples Wilcoxon test (also known as Wilcoxon signed-rank test).

**Results:**

Twenty-one patients with 2,435 h of simultaneous BT and ICP monitoring were studied. All patients reached a BT of 38 °C and experienced at least one episode of ICP above 20 mm Hg. The linear mixed-effects model revealed an association between BT above 37.5 °C and higher ICP levels that was not confirmed for lower BT. We identified 149 episodes of BT changes. During BT elevations (*n* = 79) ICP increased, whereas CPP was reduced; opposite ICP and CPP variations occurred during episodes of BT reduction (*n* = 70). All these changes were of moderate clinical relevance (increase of ICP of 4.5 and CPP decrease of 7.5 mm Hg for BT rise, and ICP reduction of 1.7 and CPP elevation of 3.7 mm Hg during BT defervescence), even if statistically significant (*p* < 0.0001). It has to be noted, however, that a number of therapeutic interventions against intracranial hypertension was documented during those episodes.

**Conclusions:**

Patients after TBI usually develop BT > 38 °C soon after the injury. BT may influence brain physiology, as reflected by ICP and CPP. An association between BT exceeding 37.5 °C and a higher ICP was identified but not confirmed for lower BT ranges. The relationship between BT, ICP, and CPP become clearer during rapid temperature changes. During episodes of temperature elevation, BT seems to have a significant impact on ICP and CPP.

## Introduction

The injured brain is extremely sensitive and vulnerable to body temperature changes [1, 2]. An increase in temperature leads to an increase in cerebral metabolism, with augmented cerebral blood flow, and a concurrent increase in cerebral blood volume. If the compensatory mechanisms are exhausted, this high cerebral blood volume [3] may raise ICP [4]. In an experimental model of brain contusion, hyperthermia (39 °C for 3 h) caused enlargement of the contusion volume and had a negative effect on outcome [5].

High body temperature can also worsen the cerebral ischemia. In experimental models of brain ischemia, hyperthermia increased the release of glutamate [6] and the extent of tissue damage [7]. Even though TBI is a different pathology from acute ischemic stroke, there is evidence [8] that abnormalities in flow metabolism coupling and areas of true ischemia are fairly common in patients with TBI.

Most patients with moderate and severe TBI experience hyperthermia during their intensive care unit (ICU) stay [9–11] and are therefore exposed to the deleterious effects of increased temperature on ICP, brain metabolism, and risk of ischemia.

A substantial proportion of experimental and clinical evidence on the interplay between hyperthermia and the brain is based on temperature measured outside the brain, either with sensors measuring temperature in the bladder [12–14], rectum [12, 13, 15–17], esophagus [12], pulmonary artery [13, 18], and jugular vein [15], which are collectively indicated as core temperature (CT), or placed externally (axillary [13] and tympanic [12] temperatures).

Unfortunately, temperatures measured outside the brain may markedly underestimate the BT, especially when it rises [18]. The mean difference between BT and CT is 0.3–0.4 °C, but it may be significantly higher during the development of pyrexia (up to 2.6 °C) depending on several factors [15, 18–21]. Nevertheless, direct BT monitoring in patients with brain injuries is rarely used [22].

We consulted a centralized data collection covering several centers in Europe [23] to obtain information on simultaneously monitored BT and ICP. The aims of this study were the following:

1. To provide a general description of BT, ICP, and CPP in a limited sample of patients with TBI.
2. To clarify the relationships between BT, ICP, and CPP during acute BT changes.

## Methods

### Patient Population

Of the 2,138 patients in the ICU in the Collaborative European NeuroTrauma Effectiveness Research in Traumatic Brain Injury (CENTER-TBI) data collection, a subgroup of 277 patients had high frequency digital signals from ICU monitoring (full waveform resolution at sampling frequencies at least 100 Hz, provided by the patient monitors) and was named High Resolution (HR) CENTER-TBI substudy (HR CENTER-TBI). These patients were enrolled in 21 centers from January 2015 to December 2017 and treated in accordance with current evidence-based guidelines for TBI [24, 25]. In total, 102 patients from this cohort had simultaneous temperature and ICP monitoring; BT was monitored in 22 of them. Hypothermia was induced in one patient and continued throughout the whole HR monitoring. Our analysis, therefore, includes 21 patients in whom ICP was measured through parenchymal probes.

Data collection in the CENTER-TBI study adhered to ethical standards; medical ethical committees of all participating centers approved the study. Informed consent was obtained in accordance with local regulations [23].

### Data Collection and Processing

Data were collected using ICM + software (Cambridge Enterprise Ltd, Cambridge, UK, http://icmplus.neurosurg.cam.ac.uk), Moberg CNS Monitor (Moberg Research Inc, Ambler, PA, https://www.moberg.com), or both. Arterial blood pressure (ABP) was obtained through arterial lines connected to pressure transducers. ICP was acquired from an intraparenchymal strain gauge probe (Codman ICP MicroSensor; Codman & Shurtleff Inc, Raynham, MA) or parenchymal fiber optic pressure sensor (Camino ICP Monitor; Integra Life Sciences, Plainsboro, NJ, https://www.integralife.com/). BT was measured through invasive parenchymal monitoring (Licox probe; Integra, Licox Brain Oxygen Monitoring System, Plainboro, NJ), usually placed in the frontal lobe. Signal processing was done with ICM + software. Signal artifacts were removed partially automatically and partially manually. The whole process of HR CENTER-TBI signal acquisition and data processing is described in previous publications [26, 27]. Because none of the included patients had an induced hypothermia, BT lower than 36.0 °C was excluded from the final analysis.

Parallel to the digital HR monitoring, information on specific therapeutic interventions (such as osmotherapy, changes in sedation, suctioning, etc.) was recorded and synchronized to the corresponding monitored variables using ICM + . Interventions and their timing were subsequently extracted using HDFView Software (The HDF5 Group, https://www.hdfgroup.org) and analyzed.

CT was not recorded at high frequencies in the selected patient cohort, and thus the information on maximal daily CT (with the probe located in rectum, bladder, esophagus, tympanum, and nasopharynx) together with the epidemiological data were accessed using a bespoke data management tool, Neurobot (http://neurobot.incf.org) developed by the International Neuroinformatics Coordinating Facility (INCF; www.incf.org), vs. 2.1. Analysis was done using the files available from July 2019.

### Statistical Analysis

Data are summarized as mean and standard deviation or as median and interquartile range (IQR). For the general description of BT, ICP, and CPP, colored maps were plotted (with per-minute data presented on a color scale) for the first 7 days of HR monitoring. Only simultaneous recordings of ICP and BT were analyzed, and missing values were excluded from the analysis.

CT was recorded daily by the investigators as the maximum and lowest temperature measured during a 24-h interval. To provide a comparison between BT and CT, maximum daily CT was compared with the highest BT recorded during the corresponding monitoring day.

To examine the relations between absolute values of six BT ranges (< 36.4, 36.5–36.9, 37–37.4, 37.5–37.9, 38–38.4, and > 38.5 °C) and ICP, we used a generalized mixed-effects linear model, with a random intercept per patient (that accounts for the repeated measurements in single patients). For the model, the per-minute values of BT and ICP were used. To correct for potential confounders, the mixed model was adjusted for TBI severity using poststabilization motor Glasgow Coma Scale ratings and pupil response [28]. For every BT group, the previous BT level was used as a reference group. *p* values were extracted to determine the significance of differences between ICP and BT groups. The figure was plotted using BT < 36.4 as the reference group.

To assess the effects on ICP and CPP of BT changes over time, episodes of BT elevation and reduction were manually selected according to the following criteria: elevations or reductions of at least 0.5 °C lasting from 15 min to 3 h. A maximum of five episodes was identified in each patient. BT elevation and reduction episodes were analyzed separately.

To assess the significance of ICP and CPP changes in response to BT, the baseline and end episode ICP and CPP within an episode were compared using the paired samples Wilcoxon test (also known as Wilcoxon signed-rank test). A *p* value < 0.05 was considered significant. ICP more than 20 mm Hg was defined as high ICP (hICP), and CPP below 60 mm Hg was used as an indicator of low cerebral perfusion. To define potentially harmfully high BT level, we used a threshold of BT of 38 °C.

All statistical analysis was done using R (“R: A language and environment for statistical computing.” R Foundation for Statistical Computing, Vienna, Austria. https://www.R-project.org/).

## Results

### Patients’ Characteristics and Monitoring

The study comprised 21 patients from four centers with simultaneous BT and ICP HR monitoring. Details of their baseline characteristics are given in Table 1. There were 18 men, with a median age of 50 years (IQR 36–55). Monitoring was established within 2 days after ICU admission; a total of 3,483 monitoring hours (median per patient 123; IQR 84–214 h) were analyzed. The final analysis included 2,435 h of monitoring (after excluding missing values) with simultaneous ICP and BT measurements.

**Table 1:** Patients’ general characteristics

Age	Sex	GCS (after stabilization in the ED)	Pupils	Marshall classification	Decompression	Duration of simultaneous BT and ICP monitoring (h)	Duration of whole HR monitoring (h)	GOSE at 6 months
23	M	8	Both reactive	III	Yes	159	169	6
70	M	10	Both reactive	VI	Yes	28	162	1
31	M	9	–	VI	No	110	115	–
25	M	4	Both reactive	II	No	104	123	–
53	M	8	–	–	Yes	19	39	1
17	M	7	Both reactive	II	No	112	120	7
55	M	15	Both reactive	VI	No	81	84	5
48	F	14	Both reactive	II	No	77	98	5
50	M	12	Both reactive	I	No	263	307	1
32	M	4	Both reactive	III	No	36	62	–
71	M	11	Both reactive	III	No	138	144	1
46	M	7	One reactive	–	No	352	583	5
37	M	6	–	VI	No	168	214	1
51	M	3	Both reactive	VI	Yes	92	105	5
44	M	8	Both reactive	–	Yes	51	69	3
69	M	10	–	VI	No	58	419	1
66	F	8	Both unreactive	–	No	88	133	1
55	F	8	Both reactive	II	No	218	245	–
36	M	7	Both reactive	VI	No	35	36	5
50	M	3	Both reactive	VI	No	211	219	1
69	M	8	Both reactive	II	No	35	37	5

This table shows patients’ main baseline characteristics. An en dash indicates missing data

BT, brain temperature, ED, emergency department, GCS, Glasgow Coma Scale, GOSE, Glasgow Outcome Scale Extended, HR, high resolution, ICP, intracranial pressure

Twenty of 21 patients had daily maximum CTs recorded, for a total of 93 ICU days during which both BT and CT were measured.

### BT, ICP, and CPP

**Fig. 1:**
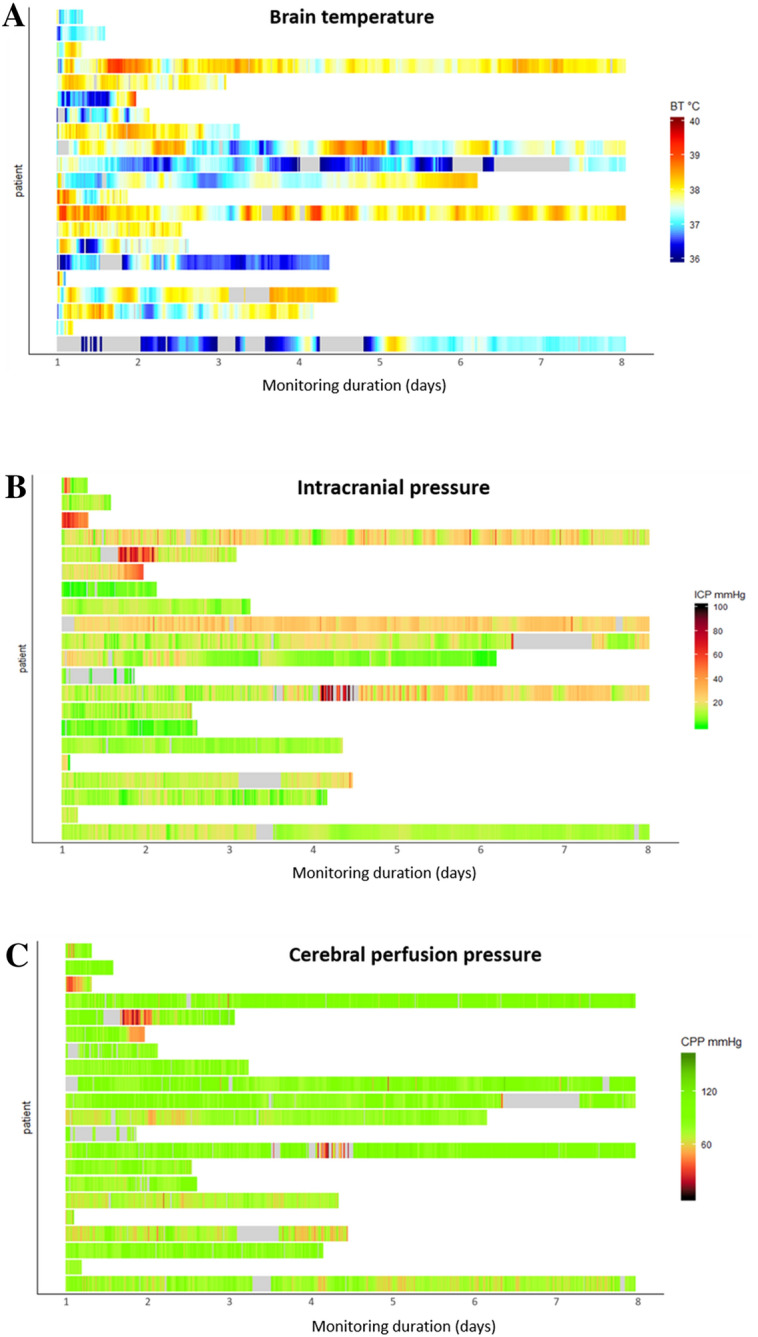
BT (**a**), ICP (**b**), and CPP (**c**) during the first 7 days of monitoring. These color maps show every per-minute average, employing a color scale with gray indicating missing values. BT, brain temperature, CPP, cerebral perfusion pressure, ICP, intracranial pressure

**Fig. 2:**
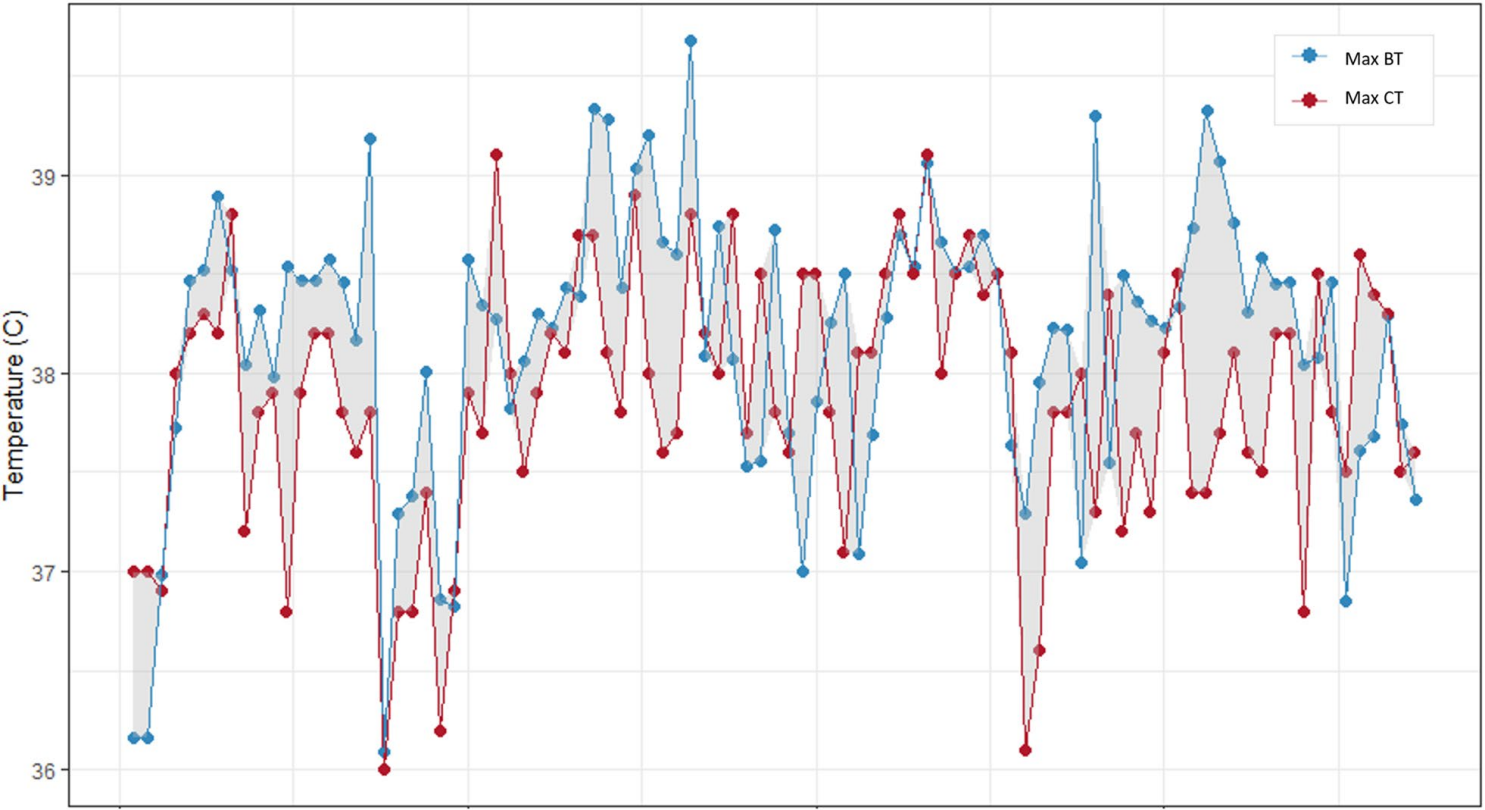
Comparison between maximal daily BT with the corresponding maximal CT. The image represents 93 available comparisons of maximal daily BT (from HR data) with the corresponding daily maximal CT (recorded manually) in 20 patients. BT and CT dots are connected by the line but, in fact, represent single measurements. In the majority of cases, BT (blue dots/line) are higher than CT (red dots/line) (*n* = 63, 68%). Maximal difference between BT and CT ranged from − 1.5 to 2 °C. BT, brain temperature, CT, core temperature, HR, high resolution, Max, maximal

The median BT for all the patients was 37.6 °C (IQR 37.3–37.9), with the lowest value of 36.0 °C reached in five patients and the highest, 39.7 °C, reached in one. All patients reached a BT of 38 °C or higher during the monitoring. BT varied widely among the patients. Figure 1a presents the BT color map during the first week of monitoring. BT maximum daily values of 38 °C or higher were observed during 65 days of matched (BT and CT) monitoring (70%). CT maximum daily temperatures exceeded that threshold only in 46 days of matched recording (49%) (Fig. 2).

Median ICP was 13 mm Hg (IQR 11–20). During approximately a third of monitoring time (31%), ICP reached > 20 mm Hg. All patients experienced at least one episode of hICP (Fig. 1b). Median of mean ABP (mABP) was 93 mm Hg (IQR 87–104). Median CPP was 83 mm Hg (IQR 79–86). All patients suffered episodes of CPP < 60 mm Hg (Fig. 1c); these episodes were short lasting, with only 5% of CPP recording time under the threshold.

**Fig. 3:**
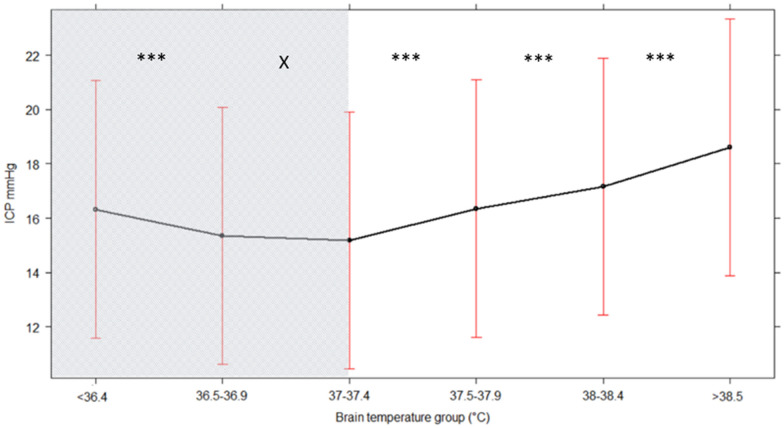
Generalized linear mixed model effects on ICP of six BT ranges. Generalized linear mixed model, including ICP (mm Hg) as dependent variable (predicted values with 95% confidence interval) in six ranges of BT (°C) as independent variable. BT < 36.4 °C was taken as the reference group. The gray area indicates the values below the physiological BT range, which are likely to depend on active treatment. The asterisks indicate the following *p* values: *X*, < 1; * < 0.01; ** < 0.001; and *** < 0.0001. BT, brain temperature, ICP, intracranial pressure

The linear mixed-effects model examined the interplay between six ranges of BT and ICP (Fig. 3). BT above 37.5 °C was associated with significantly higher ICP (*p* < 0.001). For lower BT ranges (< 36.4 °C and 36.5–36.9 °C), this association was not confirmed.

### BT Elevation/Reduction Analysis

**Fig. 4:**
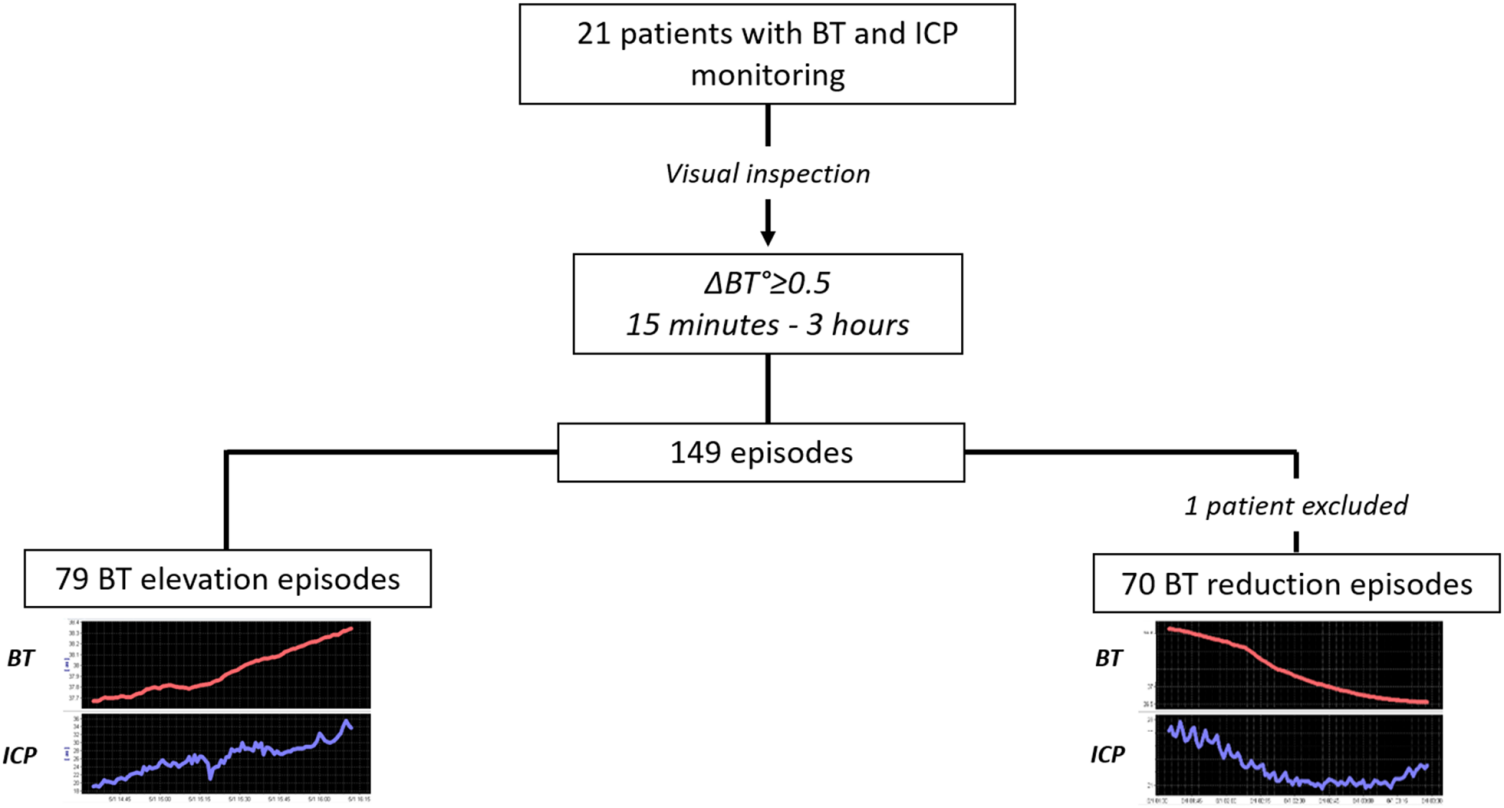
BT elevation/reduction episodes. BT, brain temperature, ΔBT, change in brain temperature, ICP, intracranial pressure

We identified 149 episodes of BT changes (at least 0.5 °C): 79 elevations and 70 reductions (Fig. 4). The total duration of all the selected episodes was 321 h (13% of HR monitoring included in the current analysis).

**Fig. 5:**
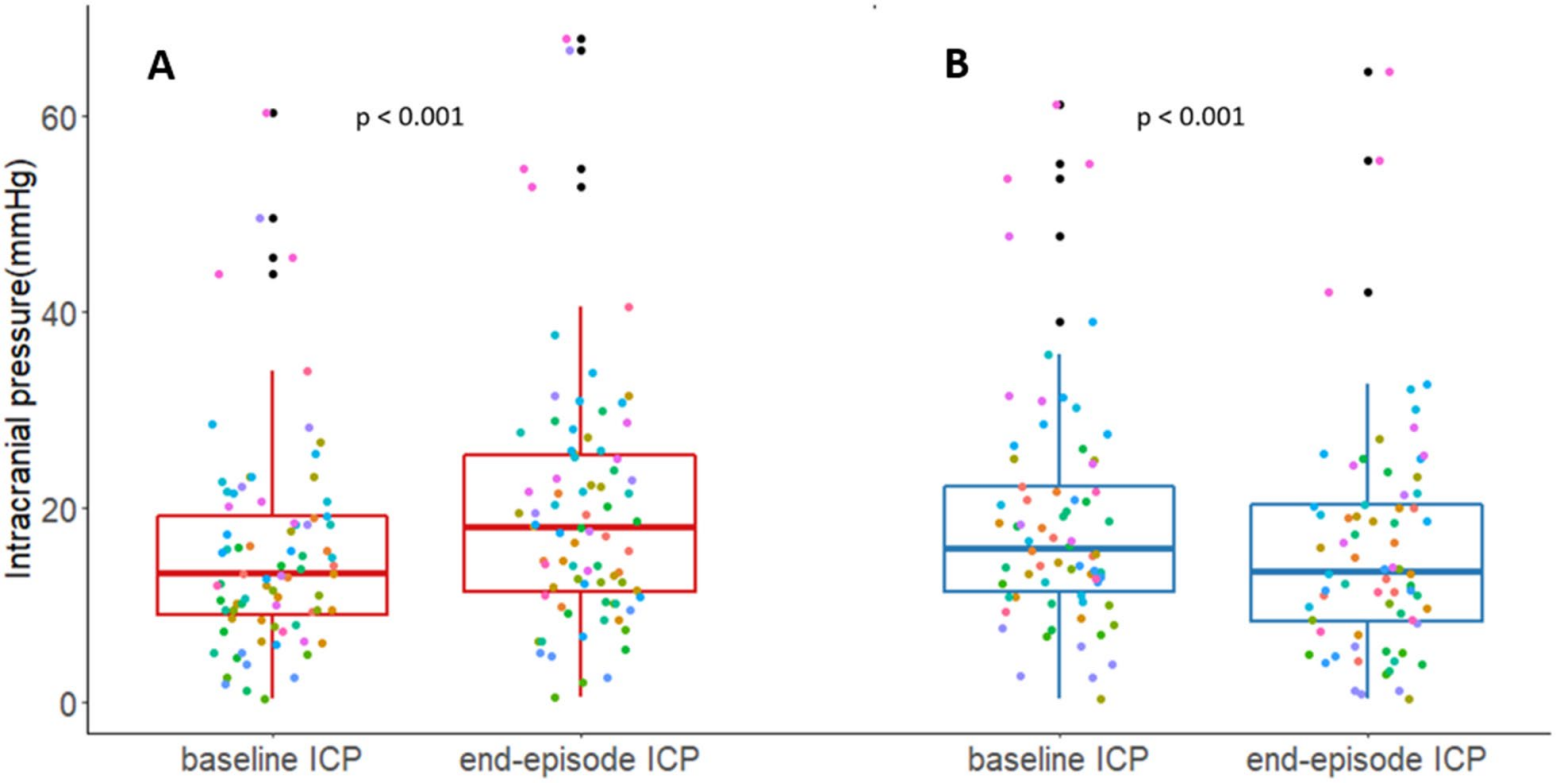
ICP response to BT changes. ICP at the beginning and end of BT episodes. **a** ICP during all the BT elevation episodes. **b** ICP during all the BT reduction episodes. Colored points represent different patients; all measurements in the same patient are the same color. *p* values for the paired samples Wilcoxon test (also known as Wilcoxon signed-rank test). BT, brain temperature, ICP, intracranial pressure

Figure 5 describes ICP patterns during BT elevation (panel a) or reduction (panel b). The median BT elevation was 0.67 °C (IQR 0.57–0.9) and the median ΔICP was 4.5 mm Hg (IQR 0.7–7.1) (*p* < 0.0001). BT reached > 38 °C in 40 episodes (51%), and hICP was observed in 33 cases (42%). During these BT increases, mABP remained constant, and consequently CPP decreased, with a median difference of 7.5 mm Hg (IQR 0.9–13.6); this change was significant (*p* < 0.0001). During 44 episodes of BT elevation, 128 active therapeutic interventions were recorded (Table 2).

**Table 2:** Interventions recorded during HR monitoring

Intervention	HR monitoring, *n*	BT elevation episodes, *n*	BT reduction episodes, *n*
Fluids	35	8	3
Osmotherapy	122	4	5
Suctioning	450	33	20
Physiotherapy	571	24	31
Sedatives	609	27	22
Vasopressors	678	32	25
Total	2465	128	106

BT, brain temperature, HR, high resolution

Starting from a median baseline BT of 38 °C (IQR 37.7–38.4) in the 70 episodes of BT reduction, there was a median decrease of 0.67 °C (IQR 0.58–0.81). ICP decreased as well, with a median reduction of 1.7 mm Hg (IQR − 1.22 to 6.03) (*p* < 0.001) (Fig. 5). Mean ABP remained constant and was accompanied by a significant (*p* < 0.005) but not clinically relevant increase of CPP (ΔCPP 3.7 mm Hg; IQR 1.96–9.8). During 41 episodes of BT reduction, 106 interventions were recorded (Table 2).

## Discussion

Fever is frequent after traumatic, ischemic, and hemorrhagic injuries [10, 29–32]. In experimental models induced hyperthermia increases the extent of the contusion volume and is associated with a worse outcome [5]. However, spontaneous fever is different from the external heating of animal heads used in the experimental setting. Moreover, the direct effect of fever on the extent of neurological injury in clinical practice is particularly difficult to separate from the impact of infection (which very often is the cause of fever and originates complex inflammatory cascades).

However, there is some indirect evidence that spontaneous fever may precipitate neurologic injury in patients with ischemic stroke and multiple sclerosis [33–35]. In subarachnoid hemorrhage, fever control is associated with reduced cerebral metabolic distress, irrespective of ICP [30]. Meanwhile, in patients with TBI, there is an association between duration and intensity of fever with worse outcome [10].

Finally, temperature elevations may lead to ICP perturbations [18] and, conversely, fever treatments (both physical and pharmacological) may reduce ICP [36, 37].

Most studies have monitored body temperature by either external or internal probes. However, existing evidence demonstrates, that BT can not necessarily be predicted from systemic temperature [38]; thus, to understand brain physiology better, direct information on BT would be of great interest, even if it requires an invasive approach [39].

To our knowledge, this is the first study to use continuous, high frequency, simultaneous monitoring of BT and ICP in patients with TBI.

The first aim was to describe the behavior of BT, ICP, and CPP and their interactions in a selected sample of patients during the first week after injury. BT showed a range of changes (Fig. 1a). All patients experienced BT higher than 38 °C, which is consistent with previous data [9, 18, 21].

Intermittent daily CT recording provided, as expected, less information on the occurrence of elevated temperatures than the more granular documentation offered by HR. According to CT maximal daily values, temperatures above 38 °C were disclosed for 46 days and a pathological BT was measured for 65 days. This finding indicate that CT may underestimate the severity of hyperthermia, as reported previously [18].

Concomitantly, ICP was generally well controlled, as reflected by a median of 13 mm Hg (IQR 11–20). However, ICP fluctuated, so each patient suffered some hICP episodes and low CPP (< 60 mm Hg).

The generalized linear mixed model gave a biphasic pattern, tending toward higher ICP with BT above 37.5 °C and the opposite below BT of 37.4 °C (Fig. 3). One possible explanation is that BT ≤ 37.4 °C depends on active manipulations, generally those used to control pathologically high ICP. Even though the physiological range of BT has not been established yet, current evidence suggests that it should be higher than 37 °C [15–21], the normal body temperature [40]. In fact, BT was generally higher than body temperature in all papers [15–21] but two [14, 29]. BT below this level is not physiological and may well depend on active treatment (i.e., intentional moderate cooling) or be a side effect of other therapies (deep sedation, myorelaxants, etc.) generally used to treat hICP. Bearing these considerations in mind, we separated Fig. 3 into two areas, showing in gray the part where we suspect the effect of treatment.

Focusing on the ICP increase with BT more than 37.5 °C, our data partially contrast with some previous reports. Four studies [18, 19, 21, 41] found no consistent relationship between BT and ICP when monitoring data were pooled and analyzed. However, the pattern we describe was found in one report of 72 patients, and in another series in which ICP was studied as a function of body rather than BT [11, 17].

The second aim of this study was to elucidate the impact of rapid BT changes (from 15 min to 3 h) on ICP and CPP. We explored 149 episodes of significant BT changes (more than 0.5 °C) and found that both ICP and CPP deteriorated when BT rose. ICP and CPP changes were significant (*p* < 0.0001) but, more importantly, they were clinically relevant, with a median ICP increase of 4.5 mm Hg, that, in 40% of the episodes has crossed the threshold of 20 mm Hg by the end of BT elevation. During these episodes active treatment for intracranial hypertension was provided, including osmotherapy and sedative and vasoactive drugs, documented by the total of 128 interventions during 44 BT elevation episodes (Table 2). It is therefore plausible to consider that therapy mitigated the actual ICP and CPP deteriorations caused by the rise in BT. In the absence of treatment, more severe ICP and CPP alterations could well result from BT increases.

Reductions of BT were studied in 70 episodes. These events too affected ICP and CPP, reducing them both slightly but significantly.

Three previous studies looked into the relationship between ICP and rapid BT changes. Two studies from our group [9, 18] suggested an association, but this was not confirmed by Hushak et al. [21]. In clinical practice, it is a common observation at the bedside that ICP can worsen during the development of fever and a recent consensus statement on TBI management suggested the correction of hyperthermia as one of the first steps for ICP control [42].

Our analysis has limitations: first, it involved a limited number of patients in few centers. Generalization of our results, therefore, would call for a larger cohort. Second, the physiopathological hypothesis linking BT to ICP and CPP is based on changes in cerebral metabolism, blood flow and content, as suggested in the Introduction. Since we did not measure these variables, our interpretation of the findings has to be considered speculative. Moreover, our study lacks the data on temperature treatments; this makes the conclusion about the natural physiological behavior of BT and ICP more complicated. Finally, our data set did not include simultaneous and continuous high frequency recording of CT and BT, which could be extremely informative; consequently, our comparison of CT and BT was restricted to a limited data set. The graphical comparison of BT and CT (Fig. 2) have some inaccuracy, and measurements of BT and CT might not correspond perfectly.

## Conclusions

BT can be monitored in patients with TBI during their time in the ICU, and temperature tends to vary widely, with frequent significant BT elevations (BT > 38 °C). A general analysis indicates that BT exceeding 37.5 °C seems to involve a concomitant rise in ICP. The relationships between BT, ICP, and CPP become clearer during rapid temperature changes. During episodes of temperature increase, BT seems to have a significant impact on ICP and CPP despite active treatment to prevent intracranial hypertension. A similar but less severe impact is seen when temperature decreases.

## Abbreviations

ICU: Intensive care unit
HR: High resolution
TBI: Traumatic brain injury
ABP: Arterial blood pressure
mABP: Mean arterial blood pressure
ICP: Intracranial pressure
CPP: Cerebral perfusion pressure
BT: Brain temperature
GSD: Standard deviation
IQR: Interquartile range
hICP: High ICP
GCS: Glasgow coma scale
GOSe: Glasgow outcome scale extended
ER: Emergency room

## Declarations

### Clinical trial registration

ClinicalTrials.gov: NCT02210221.
